# Transgenerational epigenetics in the germline cycle of *Caenorhabditis elegans*

**DOI:** 10.1186/1756-8935-7-6

**Published:** 2014-03-29

**Authors:** William G Kelly

**Affiliations:** 1 Biology Department, Emory University, Atlanta, GA 30322, USA

## Abstract

Epigenetic mechanisms create variably stable changes in gene expression through the establishment of heritable states of chromatin architecture. While many epigenetic phenomena are, by definition, heritably passed through cell division during animal and plant development, evidence suggests that ‘epigenetic states’ may also be inherited across multiple generations. Work in the nematode *Caenorhabditis elegans* has uncovered a number of mechanisms that participate in regulating the transgenerational passage of epigenetic states. These mechanisms include some that establish and maintain heritable epigenetic information in the form of histone modifications, as well as those that filter the epigenetic information that is stably transmitted. The information appears to influence and help guide or regulate gene activity and repression in subsequent generations. Genome surveillance mechanisms guided by small RNAs appear to be involved in identifying and directing heritable repression of genomic elements, and thus may participate in filtering information that is inappropriate for stable transmission. This review will attempt to summarize recent findings that illustrate this simple nematode to be a truly elegant resource for defining emerging biological paradigms.

As the cell lineage that links generations, the germline is the carrier of both genetic and epigenetic information. Like genetic information, information in the epigenome can heritably affect gene regulation and phenotype; yet unlike genetic information, the epigenome of the germ lineage is highly modified within each generation. Despite such alterations, some epigenetic information is highly stable across generations, leading to transgenerationally stable phenotypes that are unlinked to genetic changes. Studies in the nematode *C. elegans* have uncovered mechanisms that contribute to transgenerational repression as well as to the expression of genes that rely on histone modifying machinery and/or non-coding RNA-based mechanisms. These studies indicate that epigenetic mechanisms operating within the germ cell cycle of this organism filter and maintain an epigenetic memory that is required for germ cell function and can also influence gene expression in somatic lineages.

## Review

### Introduction

**Figure 1:**
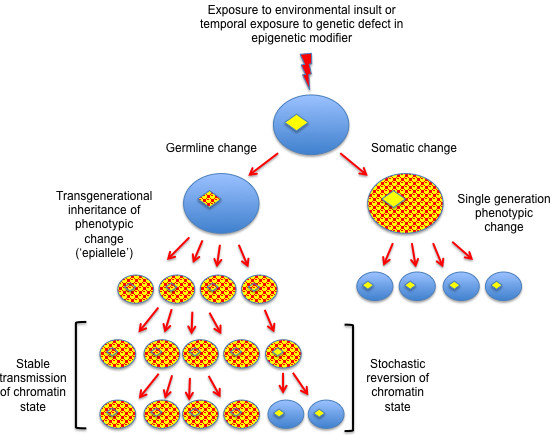
**Transgenerational epigenetic memory.** The soma (oval shapes) and germlines (diamonds) of a developing embryo are depicted. A transient environmental insult, or transient loss or ectopic activation of an epigenetic ‘modifier’ (lightning symbol) can create a change in the chromatin architecture in the genome (red pattern). If change in chromatin occurs in the developing soma (right side), it may be inherited though cell division, but the change is (usually) limited to somatic lineages. If the chromatin is altered in the germline (left side), it may become stabilized, and a phenotypic alteration in the soma or germline may be observed in the offspring for multiple generations. The ‘epiallele’ often stochastically reverts to the original chromatin structure, resulting in a reversion to the original ancestral phenotype.

The term ‘epigenetics’ was initially used to describe the constellation of developmental phenotypes that could be produced from a single genotype. The current definition of epigenetics, which seeks to encompass a very wide variety of biological phenomena, restricts this to the following example: ‘An epigenetic trait is a stably heritable phenotype resulting from changes in a chromosome without alterations in the DNA sequence’ [1]. The use of the term ‘heritable’ in the current definition can encompass mitotic stability, generational stability, or both. Thus, although stable alterations of gene expression in nondividing cells are often included in examples of epigenetic phenomena, this is stretching the current restrictive definition. Epigenetic heritability is normally less stable than genetic heritability. Whereas the reversion of a genetic change leading to a phenotype is exceedingly rare, epigenetic changes (‘epialleles’) can often be unstable, or ‘metastable’. Thus, epialleles may arise with variable penetrance within a population and may persist or disappear stochastically (Figure 1). As will be discussed below, most mechanisms linked to epigenetic processes impact chromatin structure, which indicates that chromatin structure, like DNA sequences, contains heritable information that guides gene activity. Thus, just as the genome comprises the sequence of bases in DNA, the ‘epigenome’ comprises the sum of the chromatin architecture.

The epigenome’s architecture is influenced and/or established by chemical modifications to DNA and chromatin proteins, for example, nucleosomal histones. The modifications act to attract or repel effector proteins that largely exist in multiprotein complexes and ultimately function to change the accessibility of the DNA to other complexes such as DNA and RNA polymerases and DNA repair machinery. The increasingly broad spectrum of so-called ‘epigenetic modifications’ is vast, and it has been hypothesized that different combinations of post-translational modifications to nucleosomal histone proteins can provide a ‘histone code’ that provides combinatorial cues directing specific genome activities [2]. Although controversial, it is clear that subsets of modifications, and combinations thereof, can cause or at least correlate with different states of genetic activity. Importantly, the modifications to DNA (for example, cytosine methylation and hydroxymethylation), post-translational modifications to nucleosomal histones (including acetylation, methylation, and phosphorylation), and the alteration of nucleosomes by the insertion of numerous histone variant isoforms are dynamic and therefore reversible processes. Indeed, removal activities have been characterized for most of the modifications known. This reversibility can create instability, which helps explain epigenetic metastability but creates a conflict with the hallmark of epigenetic processes - their heritability.

Although the definition of epigenetics has undergone some restriction, what is currently classified as ‘epigenetic research’ has exploded in the last decade. In the face of such expansion, it is probably useful to consider two main mechanistic components that have to exist in any epigenetic process: 1) There is an initiating event that affects activity or structural state at a locus or loci, and 2) there are subsequent processes that maintain the altered activity or state despite multiple rounds of genome replication, and these mechanisms can (or should) be separable from the initiating process(es). This holds true for both heritable gene repression and gene activation, but also for other aspects of chromosome regulation that are affected by chromatin architecture (for example, DNA replication origins, telomere stability, and centromere formation). Notably, the existence of maintenance activities solves the problem of heritability of epigenetic information described above; once established, the modifications can persist as long as maintenance activities outweigh activities that act to remove them.

The issue of maintenance has gained increased importance with the recent growing interest in ‘transgenerational epigenetic inheritance’: that is, phenotypes that are initiated by environmental changes, or transient disruption of activities linked to epigenetic regulation, that persist for multiple generations after the normal environment or activity is restored. Just as alterations in DNA sequence at a promoter affecting gene expression can be inherited by multiple generations, it is proposed that alterations in chromatin architecture (created or guided by epigenetic mechanisms) are likewise heritably stable and can affect gene expression for multiple generations. Although this is an attractive idea, and numerous phenomena have been described, the actual data supporting such inheritance currently tends to be correlative. Changes in modifications to DNA and/or post-translational modifications to histones are often observed, but the mechanistic association between the initiating events and these changes or the establishment and maintenance of a (meta-)stable phenotype is not always well established [3]. Moreover, it is often difficult to completely rule out that the heritable effect is not due to indirect genetic, rather than epigenetic, causes. There are also numerous barriers to epigenetic changes surviving multiple generations in sexually producing organisms, including the aggressive epigenetic reprogramming mechanisms that occur during gametogenesis, after gamete fusion, and during germline development within each generation. All of these barriers exist in the germline, which is the tissue through which any heritable epigenetic alterations must pass.

### Germline memory equals transgenerational memory

The concept of an immortal germline, that is, that a continuous cell lineage connects all generations, overlaps with the concept of transgenerational epigenetic memory. Epigenetic alterations that are inherited through multiple generations must both initiate and become stabilized in the germline (Figure 1). There is growing evidence that, similar to genetic mutations, epigenetic alterations that occur within the germline in one generation can be stably inherited by subsequent generations (discussed in [3]). These alterations, which cannot be explained by single generation maternal effects or cytoplasmic inheritance, have the potential to influence transcriptional activity in both the soma and germline of the descendants. As mentioned, epigenetic information is by its nature metastable and requires maintenance mechanisms for its persistence both within and between generations. This maintenance is not an easy task. The germline undergoes developmental processes that involve dramatic alterations to chromatin structure, such as those observed in meiotic chromosomes and during spermatogenesis, and any transgenerational epigenetic memory can neither interfere with, nor be dramatically altered by such intragenerational germline events. In addition, epigenetic reprogramming mechanisms greet the gamete genomes at fertilization, so information incorporated into the parental ‘epigenomes’ must avoid erasure or modification by these mechanisms in order to persist in the germline and/or soma, of the offspring [4]. The nature and transgenerational stability of epigenetic information, the mechanisms that maintain or erase this information within and between generations, and the processes that provide discriminatory targeting for maintenance and/or removal are under intense investigation, and are still poorly defined in any organism.

Studies using the nematode *C. elegans* have identified a number of mechanisms that contribute to the establishment of epigenetic patterns that are transmitted between multiple generations, as well as mechanisms that limit this transmission via epigenetic reprogramming. *C. elegans* lacks DNA methylation, a mechanism with well-characterized maintenance processes that has the strongest correlation with heritable epigenetic states (for example, [5,6]). However, DNA methylation and its heritable maintenance in those organisms where it occurs is influenced, if not regulated, by histone modifying activities [7]. As will be discussed in this review, recent studies in *C. elegans* have shown that defective regulation of histone modifications, particularly histone methylation, correlates with heritable phenotypes. In addition, histone methylation ‘maintenance’ activities have been identified that appear to contribute to the transgenerational stability of this information. Importantly, some of these activities have been implicated in transgenerational epigenetic phenotypes associated with complex somatic processes such as aging. Furthermore, there has emerged a distinct role for RNAi-related mechanisms that contribute to repressive epigenetic memory that persists across many generations. All of these processes occur within the germline, an ‘immortal’ lineage that engages in recurring developmental cycles across generations. In this review I will summarize the data that illustrates how *C. elegans* has become a useful model for transgenerational epigenetic processes and discuss what studies of this organism imply about how the epigenetic information that guides genome function may be established, maintained, and filtered through the germline in other organisms. As the germline is ground-zero of all transgenerational processes, it is first important to discuss the germline in the context of its ‘immortality’.

### The germline cycle

Unlike somatic lineages, the germ lineage contributes directly to subsequent generations and links all generations. Because of its connectivity with potentially infinite generations, the germ lineage has been classically, and perhaps romantically, termed an ‘immortal’ lineage. Thus stable or even metastable alterations to the germline epigenome, like any genetic alteration, have the potential to affect the phenotype of multiple generations. As with somatic lineages the germline also engages in tissue- and sex-specific developmental programs within each generation. Unlike somatic development, however, the developmental program occurring in one generation is directly linked to the reiteration of that program in the next generation, creating a repeating, cyclical lineage that has transgenerational continuity within the species. The germline, once established and populated during embryogenesis, exits proliferative stages and the cells enter meiosis, a germline-specific process that is similar, but not identical, between the sexes [8]. The postmeiotic differentiation of *C. elegans* gametes, especially spermatogenesis, rivals most somatic differentiation processes in terms of complexity and uniqueness of phenotype [9,10].

The fact that germ cells differentiate is interesting because the germline, by virtue of its direct contribution to total generative capacity at each generation, is considered a totipotent lineage. In somatic tissues, terminal differentiation is accompanied by substantial epigenetic programming that cements commitment to specific phenotypes, thus differentiation is usually associated with loss of pluripotency. Gamete differentiation may thus also establish epigenetic programming that is incompatible with pluripotency. The germline may be protective of its pluripotency during development, however, and the existence of parthenogenic modes of development (that is, normal development from unfertilized ova) in some organisms suggests that, at least for oocytes, loss of pluripotency during germ cell differentiation is not always the case [11]. Parthenogenesis notwithstanding, gamete development is accompanied by significant epigenetic programming that has the potential, if stabilized, to impact developmental events in subsequent generations.

Perhaps to counteract the epigenetic consequences of gamete differentiation, dramatic epigenetic ‘reprogramming’ events are observed in the gamete pronuclei after fertilization, and these events have been shown to be essential for normal development. In mammals, this consists of changes in heterochromatin organization and genome-wide DNA demethylation [4,12]. This conserved requirement for reprogramming, and the lethal consequences to its disruption, indicates that at least some epigenetic content carried in the gamete chromatin is detrimental to proper development, and its modification or removal is required to ‘reset’ the respective genomes to the pluripotent state.

Not all epigenetic content (for example, DNA methylation and histone modification patterns, *etcetera*) in gametes is removed, however, which also indicates that the process is discriminatory. In mammals, there is both active enzymatic removal and passive (for example, DNA replication without maintenance) loss of cytosine methylation, as well as conversion of the predominant methyl modification, 5-methylcytosine, to oxidized forms, such as 5-hydroxymethylcytosine [13]. In *C. elegans* zygotes, as in mammals, there are significant alterations to the genome structure, again most strikingly in the sperm chromatin, which rapidly decondenses after fertilization and prior to pronuclear fusion in many organisms [14]. Sperm decondensation is accompanied by incorporation of the histone H3 variant, histone H3.3, which is maternally provided and can become enriched in the sperm pronuclear chromatin relative to that of the oocyte [14-16]. There are also initial differences in a number of histone modifications between the male and female pronuclei prior to fusion in both mammals and *C. elegans*[14,15] (WK, unpublished work).

To broadly summarize: the gamete genomes initially meet at fertilization with significant differences in developmental histories, and these differences are reflected in their respective epigenetic contents. In the zygote, many of these differences are subjected to reprogramming/remodeling prior to pronuclear fusion. Although many parent-of-origin differences appear to be resolved prior to genome fusion, some differences clearly persist in many species as evidenced by the different epigenetic states of imprinted loci [17]. The diploid zygote’s epigenome is therefore a highly manipulated composite of the two separate epigenomes of the gametes. This begs the question of whether there can be an ‘immortal germ line’ that comprises a continuous epigenetic component, or whether there is significant discontinuity in the germ lineage that requires re-establishment of epigenetic content at each generation. At least in *C. elegans,* there is clearly information that is stable between and across generations, yet there also appears to be a ‘re-establishment’ phase required for proper germ cell development in this organism. Before clarifying this statement, I will first introduce the reader to germ cell development in *C. elegans*. I will then summarize evidence that histone modifications and the machinery that regulates them, often in concert with non-coding RNAs, contribute to a memory of either gene activation or repression that can stably impact the organism’s transcriptome for multiple generations.

### The *C. elegans* germline cycle

**Figure 2:**
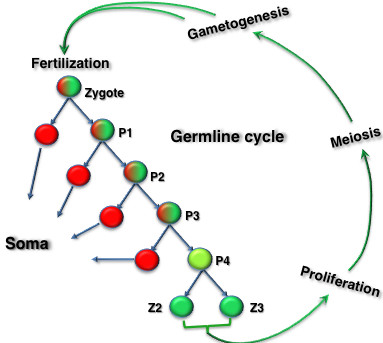
**The germline cycle in *Caenorhabditis elegans*.** The first four cell divisions after fertilization are asymmetric, with the posterior germ line precursor ‘P-cells’ (P1 to P4) inheriting germplasm (green). The anterior cells at each division contribute to various somatic lineages (red). The last division at P4 is symmetric and yields Z2 and Z3. Z2/Z3 divide after hatching, and their descendants undergo extensive proliferation and germ cell development (meiosis and gametogenesis). The cycle is repeated after the gametes fuse in the zygote.

Germline development in *C .elegans* (Figure 2) has long been considered an example of the ‘preformistic’ mode of germline development; that is, maternally-derived cytoplasmic determinants ‘pre-form’ the germline in the oocyte or early embryo (as opposed to ‘epigenetic’ or ontogenic modes in which inductive signals from surrounding cells specify the germline). In the preformistic mode of germline specification, the cells in the embryo that inherit this maternal ‘germplasm’ passively inherit germline identity. In *C. elegans,* the germplasm-enriched and sequentially produced *P-cells* are generated through asymmetric divisions in which the posterior daughter cells (Figure 2; P1 through P4) retain enrichment of the germplasm and associated factors. The anterior daughters of each of these asymmetric divisions (AB, EMS, C, D) become founder cells for multiple somatic lineages. The last P-cell, P4, divides symmetrically and both daughters, named Z2 and Z3, inherit equivalent amounts of germplasm. These daughters, Z2/Z3, undergo DNA replication and then arrest through embryogenesis and do not re-enter the cell cycle until after the embryo hatches, and even then, only if hatching occurs in the presence of food [18]. Postembryonic germline development first consists of proliferation in early larval stages to produce a germline stem cell pool from which cells enter meiosis and gametogenesis in later stages. Germline sex is determined using pathways governed by the X chromosome karyotype: larval XX germ cells exiting meiosis in the hermaphrodite enter spermatogenesis, but after the last larval molt only oocytes are produced. XO germ cells produce only sperm in late stage larvae and throughout the adult male’s life [19,20].

The P-cells are both cytologically and functionally distinct from their somatic sisters because maternal factors that inhibit RNA polymerase II (RNA Pol II), such as the CCCH Zn finger protein PIE-1, are also enriched in the germplasm (for review, see [21]). The somatic sisters of the P-cells actively destroy PIE-1 and other germplasm remnants, activate zygotic transcription, and engage in developmental paths guided by maternally provided transcription factors and cell-to-cell signaling [22,23]. The P-cells also inherit maternal transcription factors (for example, SKN-1 and Pal-1 [24,25]), that are drivers of somatic fates, and must maintain their ‘germline identity’ by inhibiting most gene expression to prevent somatic differentiation. In other words, the default fate of the P cells is somatic differentiation, not germline, a situation not entirely compatible with the passive default germline fate implied by the preformistic model. Somatic transformation of the germline in the absence of repression is similar in concept to the germline phenotype in mice lacking the repressor Blimp1/Prdm1, in which somatic factors (for example, Hox loci) are derepressed in the cells that normally give rise to primordial germ cells (PGCs), and the germline is lost [26].

As in mammals, the *C. elegans* embryonic germline therefore passes through a state with significant somatic potential that needs to be suppressed in order to establish the embryonic germline. The P-cells produce both germline and soma, and are thus presumably pluripotent. The last P-cell, P4, has no somatic descendants and, at least by lineage analysis, is restricted to germ cell fate and thus is often considered a primordial germ cell (PGC). However, the events that occur after the symmetric P4 division to produce Z2/Z3 create such completely different nuclear and cytoplasmic phenotypes that PGC specification may not be complete until the birth of these two cells.

**Figure 3:**
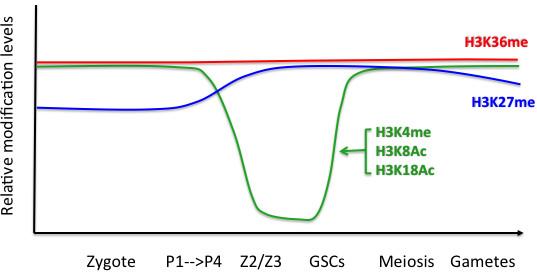
**Dynamics of histone modifications in the germline cycle.** The relative abundances of the modifications indicated are shown at different times during germ cell development in *Caenorhabditis elegans*. For simplicity, aggregate changes in the level of any specific modification at the indicated lysine (for example, H3K4me2 versus H3K4me3) are shown.

As mentioned, Z2 and Z3 undergo DNA replication but subsequently arrest in early prophase for the rest of embryogenesis (10 to 12 hrs). This arrest is reminiscent of the G2 arrest also observed during mammalian PGC development [27,28]. A remodeling of the germline epigenome occurs in Z2/Z3 that consists of dramatic genome-wide decreases in euchromatic histone modifications, including histone H3 lysine 4 methylation (for example, H3K4me2), and histone H3K8 and H3K18 acetylation (H3K8Ac, H3K18Ac; Figure 3). These events occur rapidly in Z2/Z3, and are sometimes observed to begin before or near the P4 division [29,30]. There is also an increase of the repressive modification H3K27me3 ([31]; W. Kelly unpublished work). Oddly, the disappearance of ‘active’ histone modifications and enrichment for the repressive mark H3K27me3 coincide with phosphoepitopes correlating with transcriptional elongation appearing on RNA Pol II [32,33]. This ‘activation’ of RNA Pol II is linked to the degradation of maternal PIE-1, but the connection between loss of PIE-1, the transient appearance of the RNA Pol II phosphoepitopes, and the erasure of chromatin modifications is currently unclear. The loss of H3K4 methylation does not appear to be linked to demethylase activity, and appears to involve histone replacement [34] (H Furuhashi and WK, unpublished work). The massive erasure of histone H3 modifications that occurs in Z2/Z3 in *C. elegans* may be analogous to the waves of epigenetic reprogramming that occurs during primordial germ cell specification in mice [28]. Importantly, any epigenetic information that is to be passed onto the next generation has to be resistant to the reprogramming that occurs in Z2/Z3. As discussed below, one such resistant mark, histone H3 methylated on lysine 36 (H3K36me), is an important component of the epigenetic information inherited by offspring.

### Reiterative maintenance of histone H3 methylation and epigenetic memory

**Figure 4:**
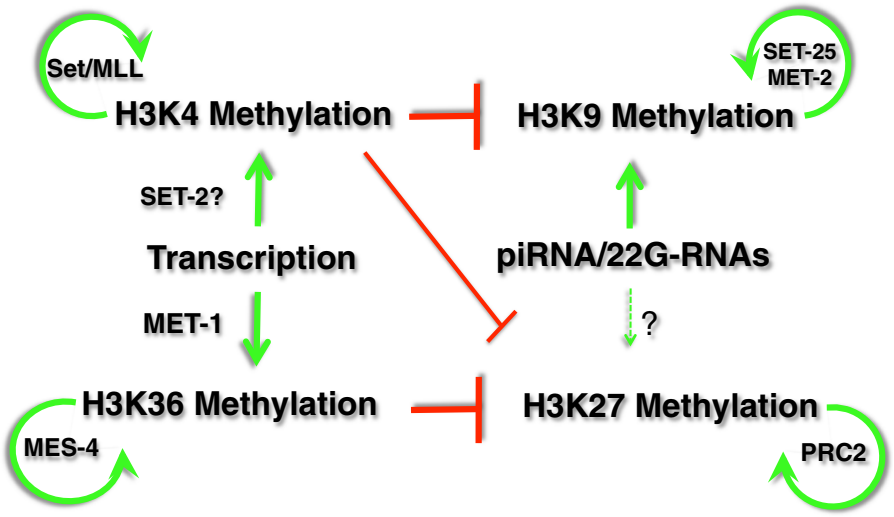
**Histone methylation establishment, maintenance, and interactions.** The mutually antagonistic relationships between histone H3 methylation on Lysines 4, 9, 27 and 36 are shown. The antagonism (red) between H3K36 and H3K27 methylation has been observed in *Caenorhabditis elegans*; the antagonism between H3K4 methylation and H3K9/H3K27 methylation has been observed in multiple organisms and is assumed to occur in *C. elegans* (for example, [37]). Straight green arrows indicate mechanisms known or suspected to establish each modification; curved green arrows indicate mechanisms known or suspected to maintain each modification.

Of the four nucleosomal histones and their modifications, methylations of histone H3 seem to play an outsized role in epigenetic memory and chromatin structural alterations. Among the modifications found on this protein (in addition to the multiple variants of H3 that are observed, such as H3.3 described above), methylation at lysines 4, 9, 27, and 36 (H3K4me, H3K9me, H3K27me, and H3K27me, respectively) are most closely associated with heritable states of transcriptional activity. In addition to the different residues, the degree of methylation at each residue can have a different distribution and biological correlation in the genome. For example, mono-methylation of histone H3 on lysine 4 (H3K4me1) is largely enriched in enhancers, whereas H3K4me2 and H3K4me3 are observed at the 5’ end of genes, with H3K4me3 more tightly associated with the transcription start site and H3K4me2 more dispersed into the gene body [35]. Likewise, methylation at the different lysines in H3 broadly correlates with either transcription activity or suppression (for more details, the reader is referred to any of the many reviews on this topic; for example, [36]). H3K4 and H3K36 methylation are usually associated with transcriptional activity or ‘euchromatin’, wheras H3K9 and H3K27 methylation are normally associated with transcriptional repression. Histone methylation on one lysine can also influence the modification status on other lysines, resulting in an interconnected and potentially self-reinforcing network (Figure 4). H3K9 and H3K27 methylation are both implicated in heritably stable forms of transcriptional silencing, with both having well-described evidence of RNA-directed targeting to genomic loci in a variety of organisms that have been studied. In contrast, the contributions to epigenetic memory of ‘active’ chromatin modifications such as H3K4 and H3K36 methylation have been less studied. Much of the evidence for the roles of these marks in transgenerational epigenetic processes has come from genetic model systems, including *C. elegans.*

### The MES-4 H3K36 methyltransferase

In yeast, a single H3K36 methyltransferase, Set2, is responsible for all H3K36 methylation [38]. This modification, while often cited as associated with gene activity, is actually a repressive modification in yeast. Set2 activity is required for the activation of histone deacetylases to decrease histone acetylation in nucleosomes after the passage of elongating RNA Pol II, which stabilizes chromatin and prevents aberrant initiation at cryptic promoters in transcription’s wake, [39-41]. Whereas Set2 is the only known H3K36 methyltransferase in yeast, most multicellular organisms have multiple H3K36 methyltransferases. The *C. elegans* genome encodes two H3K36 methyltransferases, *met-1* and *mes-4*, and these enzymes account for all detectable H3K36 methylation [33,42-44]. MET-1 is analogous to yeast Set2, in that its activity appears to be co-transcriptional [44]. MET-1 is not essential and *met-1* mutants can be maintained as a homozygous strain that is fertile but exhibits some somatic defects [42]. Interestingly, although *met-1*mutants alone have few phenotypes, *met-1* mutants in combination with mutations in *met-2* (which encodes an H3K9 methyltransferase) have a ‘mortal germline phenotype’; that is, there occurs an increased frequency of sterile offspring in each successive generation [42].

MES-4 is related to the mammalian nuclear receptor-binding SET domain proteins NSD1/2, and maternal provision of MES-4 protein is required for the germline to develop [43,45]. In contrast to MET-1, MES-4 activity is essential, but only in germ cells. Maternal provision alone of (M+) of MES-4 protein in the P-cells and Z2/Z3 is sufficient for normal proliferation and development of the hundreds of descendants of Z2/Z3 in offspring lacking any wild-type copy of the *mes-4* gene (Z-). Such offspring, denoted as ‘*mes-4* M + Z-’, are completely fertile, but produce offspring that lack maternal MES-4 (*mes-*4 M-Z-). The postembryonic germ cells in these offspring degenerate after a few cell divisions, and the animals grow to become completely sterile but otherwise largely normal adults. Maternal MES-4 protein, while initially present in all embryo cells at early stages, ultimately becomes enriched in Z2/Z3, as does the H3K36 methylation it produces [33,44]. The maternal dependence and sufficiency shows that a) MES-4′s function is critical only in the embryonic or very early postembryonic germline, and b) postembryonic germ cells no longer need significant levels of MES-4 function to generate normal numbers of functional gametes (that is, the protein is no longer detectable past early larval stages). What explains the absolute requirement for MES-4 in the transcriptionally inert embryonic germ cells, and its dispensability for postembryonic proliferation and development? The answer appears to be that MES-4 activity maintains, in the embryonic germ cells, H3K36 methylation patterns produced during transcription in the parental germline, and this pattern is required for proper postembryonic development of germ cells in the offspring.

H3K36me2/3 is incorporated into chromatin during transcription in the parental germ cells where it is enriched on autosomes but substantially diminished from X chromatin, which correlates with the diminished transcription on the X in germ cells [43]. MES-4 protein shows a similar distribution by immunofluorescence [43,44]. The H3K36me established in adult germ cell chromatin persists in the gamete chromatin, and is further maintained in the embryonic chromatin despite cell divisions and prior to the significant levels of zygotic transcription [33,44]. In the zygote, MES-4 and H3K36me2/3 remain largely absent from both X chromosomes, while a slight but observable signal is evident on the maternal X (Xm), presumably from X-linked transcription occurring during oogenesis [43] (H. Furuhashi and WK, unpublished work). Importantly, H3K36me is maintained in the transcriptionally quiescent embryonic germline chromatin and this maintenance is completely dependent on MES-4 [33,43].

In embryos lacking MES-4 the H3K36me3 level in chromatin is quickly diminished with cell division, presumably through replication-dependent dilution by incorporation of unmodified H3 [33]. H3K36 methylation in the zygote chromatin originates from what existed in the gamete chromatin. This H3K36me3 is produced by MET-1 during transcription in the adult germline, and its maintenance in the dividing embryo requires MES-4. MET-1 also produces H3K36me3 in later somatic lineages as transcription ramps up, but this mark remains absent in the P-cells and the PGCs. Conversely, in *met-1* mutants the H3K36me3 that is present in the gamete chromatin is maintained by MES-4 and follows MES-4 protein dynamics but anti-correlates with transcriptional activity. In *met-1* embryos, H3K36me3 is initially present in all embryonic cells, but becomes progressively diminished in the transcribing soma while remaining robust in the non-transcribing P-cell and PGC chromatin [33]. No H3K36me3 is detected in *mes-4;met-1* double mutants, indicating that these two MTases comprise all H3K36-directed HMT activity in *C. elegans*[44].

Genome-wide analysis of MES-4-dependent H3K36me3 patterns (ChIP-seq in *met-1* mutant embryos) and MES-4 protein distribution (ChIP-chip in wild-type embryos) revealed overlapping and surprising results: in embryos, both MES-4 and its H3K36me3 product are restricted to genes that are expressed in postembryonic germ cells [33,44,46]. Note that this gene set includes genes expressed *only* in adult germ cells and genes expressed in *all* cells. Importantly MES-4 protein is observed in loci lacking detectable RNA Pol II occupancy, and conversely, MES-4 occupancy is not observed in zygotically activated developmental genes that exhibit RNA Pol II association [44,46]. The H3K36me3 ChIP-seq pattern in *met-1* embryos is consistent with this pattern; that is, there is little detectable MES-4-dependent H3K36me3 in somatic developmental genes, which are known to be transcriptionally active in embryos [33,44,46]. MES-4 activity thus appears to be maintaining - in the embryo - the H3K36me3 patterns established during transcription in the parental germ cells. The progressive restriction of MES-4 to the embryonic germ cells similarly progressively restricts adult germline H3K36me3 patterns to this lineage.

Consistent with its proposed role as a maintenance methyltransferase, experimental evidence indicates that MES-4 predominantly adds H3K36me3 to loci where some level of H3K36 methylation already exists; that is, it reinforces pre-existing patterns of H3K36 methylation [33]. These patterns include H3K36me in genes that are expressed only in germ cells as well as genes expressed in all cell types. Furthermore, MES-4 maintenance of this pattern in the germline is essential for germ cell viability. In the absence of MES-4 protein in the parental germ cells, and hence absence of maternal MES-4 in the offspring, the MET-1 dependent patterns inherited within the gamete chromatin are quickly diluted by DNA/chromatin replication. When the mutant germ cells exit their quiescent state in larvae they die after a couple of cell divisions [45]. This suggests that in the absence of MES-4, and the H3K36me3 patterns it maintains, the transcription program is aberrant or otherwise incompatible with germ cell viability. Importantly, as will be discussed below, MES-4 H3K36 methylation can antagonizes polycomb related complex 2(PRC2)-dependent H3K27 methylation, which may contribute to *mes-4* mutant germ cell dysfunction.

Interestingly, MES-4 is required for the ectopic activation of genes that are normally only expressed in germ cells in somatic lineages [47-49]. The ectopic activation of germline-restricted genes occurs in synMuv B class mutants. This class of factors is largely made up of conserved repressor complex components, such as retinoblastoma protein, the NuRD histone deacetylase complex, heterochromatin protein 1 (HP1), and others [50]. An interpretation of the requirement for MES-4 is that the H3K36 methylation patterns maintained by residual MES-4 in the zygote must be actively counteracted or removed by repressor activities in the somatic lineages to avoid activation. MES-4 marking, in other words, is sufficient for default activation in the absence of somatic repression. Interestingly, somatic expression of germline-restricted genes also requires the worm polycomb repressor complex 2 (PRC2), which is responsible for the repressive histone modification H3K27 methylation.

MES-4 thus behaves like a maintenance methyltransferase that provides a transgenerational continuity of H3K36 methylation patterns in the germ line. The H3K36me patterns maintained by MES-4 reflect the transcriptional activity that occurred in the germ line of the preceding generations, and stable maintenance of these patterns between generations is essential for germ cell function within each generation. MES-4 may also be sufficient to maintain H3K36me3 patterns in germ cells for many generations. The H3K36me3 profile observed by ChIP-seq in *met-1* mutant embryos described above were obtained from a homozygous *met-1* strain passaged as such for many generations [33]. All experimental evidence thus far suggests that MES-4 does not add H3K36me during transcription [33,44,46]. The absence of *met-*1 thus equals the loss of co-transcriptional H3K36 methylation, yet H3K36me3 in *met-1* embryos clearly marks genes that are transcribed in adult germ cells. The H3K36me3 patterns maintained by MES-4 in these mutants may therefore have been produced during transcription in germ cells of the wild type ancestor of the *met-1* mutant many generations past. Without this reiterative marking during each generation, the pattern is largely maintained by MES-4 but with some generational weakening in absence of reiteration, thus resulting in imperfect generational maintenance of fertility in the *met-1* mutants. The MES-4′s H3K36 methylation pattern is thus essential for fertility, possibly by providing a genome architecture that promotes transcription of germ cell-expressed loci. MES-4 may function to keep promoters accessible to basal transcription factors, and it may largely accomplish this by preventing encroachment of H3K27 methylation.

### The yin and yang of MES-4 and PRC2

Recent evidence indicates that one role of MES-4 activity is required to limit the spread of the repressive modification H3K27me3 into germline-expressed genes [46] (Figure 4). H3K27 methylation in *C. elegans* is largely mediated by the MES-2/-3/-6 proteins, the worm version of polycomb group repression complex 2 (PRC2) [31]. Like mutations in *mes-4,* mutations in *mes-2, mes-3,* or *mes-6* cause maternal-effect sterility and all are likewise maternally required and sufficient [31,45]. ChIP-chip analyses of H3K36me and H3K27me in early embryonic chromatin shows that these marks, which mostly reflect their inherited patterns, are strikingly mutually exclusive [46]. H3K36me is enriched almost exclusively in the bodies of genes expressed in the germline, as previously demonstrated [33]. H3K27me, on the other hand, is non-overlapping with H3K36me and is more broadly distributed, with particularly widespread distribution on the X chromosome. The mutual exclusion of H3K36me and H3K27 methylation appears to be a consequence of antagonism between these processes: depletion of H3K36me in germline-expressed genes in *mes-4* mutant embryos allows encroachment of H3K27me into these genes from adjacent regions [46]. This antagonism probably sheds light on the profound sterility and necrotic germ cell death in *mes-4* embryos. Encroachment of H3K27me3 into germ cell-expressed loci could result in repression of many germ cell-essential loci. Spread of PRC2 activity into normally active loci may also dilute PRC2-mediated repression of it normal targets, resulting in what may be a toxic transcriptome in *mes-4* germ cells.

As in *mes-*4 mutants, the ectopic expression of germline genes in soma observed in Rb-repressor mutants is also reduced in PRC2 mutants [47-49]. MES-4 and PRC2′s antagonistic activities may maintain a heritable epigenomic architecture that, in the absence of active repression, is conducive for expression of germline-expressed genes in any tissue. Interestingly, MES-4 itself is a target of Rb-mediated repression in the soma, which may help to further dilute germline epigenetic memory in dividing somatic cells [51].

**Figure 5:**
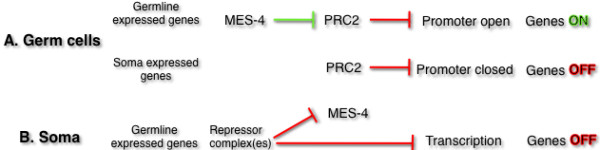
**MES-4 and PRC2 regulation of gene expression in germline and soma.A**. In genes normally expressed in germ cells (top), MES-4 activity is required for proper activation, and this is hypothesized to result from its antagonistic inhibition of PRC2-mediated repression. In genes normally expressed in soma (middle), PRC2 prevents MES-4 modification of these genes leading to a default repressed state. **B**. In somatic tissues (bottom), residual MES-4 activity marks genes for activation, but repressor complexes such as Rb and NURD (see text) normally prevent this activation in somatic lineages. MES-4 itself is also repressed in somatic lineages, presumably to reinforce this repression.

These observations *in toto* suggest that a heritable epigenetic template generated by transcription-dependent H3K36 methylation in adult germ cells, which can persist in gamete chromatin and is maintained in the zygote by MES-4 activity, helps prevent encroachment of a repressive chromatin by PRC2-mediated H3K27 methylation in the offspring. This may help to delineate genes that can and cannot be expressed in germ cells (Figure 5). The H3K36 methylation pattern maintained by MES-4 within each generation may be stably maintained for dozens of successive generations without a need for transcription-dependent reiteration at each generation for significant fertility to be retained. Within each generation, however, defective transmission of the previous generation’s pattern through the embryonic germline is immediately catastrophic. Preventing germline activation in the soma appears to require targeting these genes for repression via mechanisms involving a subset of synMuv B factors. Interestingly, these repression mechanisms do not target all genes expressed in germ cells - just those expressed solely in germ cells. Genes expressed in all cells, which are also marked by MES-4, must somehow escape this repression, but how the selectivity is achieved is unclear.

The apparent ‘default expression’ patterned by the MES-4/PRC2 antagonism is interesting, as both H3K36me andH3K27me are not noticeably affected by the observed reprogramming mechanisms, described below, that are active in Z2/Z3, even though multiple modifications that correlate with gene activation are significantly reduced. H3K36me and H3K27me may either be resistant to the reprogramming mechanisms, or the enrichment for the MES-4 and PRC2 proteins in these cells counteracts reprogramming through continual re-establishment. Indeed these modifications would have to persist in germline chromatin if a transmittal of a stable H3K36me/H3K27me pattern was instructive for germline gene expression in subsequent generations. Interestingly, another modification implicated in epigenetic memory, methylation of lysine 4 on histone H3 (H3K4 methylation), is noticeably depleted during Z2/Z3 reprogramming. Interestingly enough, several studies have implicated H3K4 methylation and the machinery that provides this modification as contributing to transgenerational phenotypes, both in the germline and the soma.

### The SET/MLL complex and H3K4 methylation

Methylation of histone H3 on lysine 4 (H3K4me) is normally associated with transcriptional activity and has been implicated in transgenerational epigenetic mechanisms. Ng and Gurdon working in *Xenopus* showed that H3 lysine 4, and presumably its methylation status, impacts the stability of epigenetic memory during reprogramming in somatic nuclear transfer experiments [52]. H3K4 methylation coincident with H3K27 methylation has been defined as a ‘bivalent’, or ‘poised’ marking of early developmental loci observed in mammalian gametes and embryonic stem cells, and has been proposed to contribute to cross-generational totipotency of the germline [53-57]. Studies using *C. elegans* strains carrying mutations in histone H3K4 demethylases indicate that this modification can be stably inherited for multiple generations through the germline. Mutations in *spr-5,* which encodes a *C. elegans* homolog of the H3K4me2 demethylase LSD1, cause a ‘mortal germline’ phenotype; that is, sterile offspring arise with increasing frequency at each successive generation [34]. Transcription profiling across many generations revealed an increase in transcripts from genes expressed during spermatogenesis that correlated with a transgenerational increase in H3K4me2 levels in the promoters of these genes [34]. In late generations, spermatogenesis-enriched gene expression levels plummeted yet the H3K4me2 levels continued to climb. The generational increase of H3K4me2 despite decreased expression indicated that the H3K4me2 increase in the promoter chromatin was not necessarily tied to ongoing transcription. These results indicate that H3K4 methylation, if not removed from some germline-expressed loci within each generation, can be stably inherited and additively increase with each subsequent generation through maintenance mechanisms that may not require active transcription. Furthermore the mortal germline defect suggests that correct patterning of heritable H3K4 methylation in any generation is required for normal transcriptional regulation in subsequent generations.

Similar to H3K36 methylation, H3K4 methylation has both transcription-coupled and transcription-independent mechanisms. The latter may be used to prepare genes for activation, as in ‘bivalent loci’ observed in pluripotent cells [53]. These are developmental loci that are enriched in both H3K4me and H3K27me, and are thus thought to be inactive but ‘poised’ for activation during early developmental decisions. Also like H3K36me, transcription-independent H3K4 methylation requires mechanisms for its maintenance. The enzyme responsible for all H3K4 methylation in yeast is Set1, which acts in a complex called COMPASS (complex proteins associated with Set1p) [58]. COMPASS and its components are highly conserved in other organisms, including mammals, where it is called the MLL complex, named after the MLL (mixed-lineage leukemia) family of histone methyltransferases (for example, [58]). In addition to the MLL or Set1-like histone methyltransferases, other highly conserved complex components include Wdr5, Ash2l, RbBP5, Cfp1, and Dpy30 [58]. Homologous proteins are found in *C. elegans* and are presumed to similarly function in complex(es) that have been referred to as Set1/MLL complexes, since it is not clear there is a single complex [59-61]. Mutation or depletion of several of the conserved MLL complex homologs in *C. elegans* causes a general, widespread depletion of H3K4me2 and H3K4me3 in all cells in the early embryo, including the transcriptionally inert P cells [59-61]. An additional H3K4 methyltransferase activity that remains active at later stages in the Set/MLL mutants appears coupled to active transcription [60]. The identity of the transcription-dependent H3K4 methyltransferase is currently unclear, although a putative H3K4 methyltransferase SET-16, is thought to play a role [62].

H3K4 methylation in the early embryo, while dependent on Set1/MLL activity, seems to be largely independent of transcription. Depletion of RNA Pol II does not cause extensive loss of H3K4me2/3 in early embryos, suggesting that the H3K4 methylation in the early embryonic blastomeres can be largely transcription independent [60]. Indeed, the maintenance of H3K4me2 levels in the dividing P cells, which lack RNA Pol II activity, supports a model that H3K4 methylation in the early embryo is largely due to maintenance of this modification, rather than transcription-dependent incorporation. As with MES-4 dependent H3K36me, MLL-dependent H3K4me in embryos appears to be maintaining the patterns inherited through the gametes [14,60]. In contrast to the complete sterility observed in *mes-4* mutants, however, mutations in MLL components initially cause only a decrease in the size of the postembryonic germline stem cell pool [60]. A partial mortal germline defect, however, is observed in later generations [59,60].

### H3K4me patterns established in adult germ cells are transmitted to the offspring

MLL-dependent/transcription-independent H3K4 methylation is substantial in the adult germline, but as with H3K36 methylation co-transcriptional H3K4 methylation also occurs and contributes to epigenetic information that is inherited by the offspring. The heritable stability is most clearly demonstrated by the sex-specific epigenetic profile of the X chromosome. The *C. elegans* X chromosome is depleted of most genes that are expressed in the germ cells of both sexes [63,64]. This X chromosome bias is even more obvious for genes required during spermatogenesis: of over 40 mutants with spermatogenesis defects, none are X-linked (S. L’Hernault, personal communication). As there are only five autosomes and one sex chromosome in *C. elegans*, this absence of sperm-expressed genes is a strikingly distorted genomic distribution. In contrast, there are many X-linked genes that activate after meiosis and are expressed during oogenesis [64]. Thus, the X chromosome has low transcriptional activity during meiosis in both germline sexes, but becomes highly active during female gametogenesis. As a consequence, while transcription-dependent H3K4 methylation is continuously low on the X chromosome in all male germ cell stages, in female germ cells H3K4me in X chromatin substantially increases during oogenesis. This difference in chromatin marks on the X’s, which are consequences of their respective difference in transcription activity, creates a chromatin memory that persists into the next generation.

The sex and chromosome bias in transcription-related H3K4 methylation is retained in gametes. The X chromosomes in oocytes are largely indistinguishable from autosomes when assayed for H3K4me2, since this mark increases on the X during oogenic transcription [64]. In contrast, the X chromosome in haploid sperm remains easily distinguished by its severe depletion of H3K4me2 relative to autosomes, which retain abundant H3K4me2. Although protamine-like proteins have been identified in purified sperm [65], there is relatively little depletion or replacement of histone H3 in *C. elegans* haploid sperm chromatin during spermatogenesis [14]. The X chromosome, unlike the autosomes (and the X in later oogenesis), also has little evident replication-independent replacement of H3 by variant histone H3.3 during male meiosis, presumably due to a marked decrease in transcription-coupled histone replacement [14,66]. Thus, when the sperm and egg chromatin meet at fertilization, they carry histones and histone modification patterns that, at a low resolution, match the patterns they accumulated during meiotic and gametogenic transcription; that is, low H3K4me and H3K36me on the sperm X chromosomes, and an abundance of both marks on the autosomes. Importantly, retention of histones and their marks in sperm is observed in other organisms, including many invertebrate and vertebrate species [67]. Zebrafish sperm chromatin largely retains histones and exhibits little if any protamine-like replacement [54]. It is important to note that although mammalian sperm are largely depleted of histones through protamine replacement, some studies have shown that some nucleosomes with modified histones may be retained at interesting loci, including genes expressed during spermatogenesis and genes that encode developmental transcription factors [55-57]. The latter class carry the bivalent marking of both H3K4 and H3K27 methylation discussed above.

As soon as the *C. elegans* sperm chromatin enters the oocyte there is a substantial influx of histone H3.3 (detected by H3.3 tagged with GFP) into the chromatin of all chromosomes, including the sperm X [paternal X or Xp [14]. After pronuclear fusion and cell division, all chromosomes including the Xp become noticeably enriched in H3K4 histone H3 unmodified at K4. An antibody that can discriminate between H3.1 and H3.3 shows little H3.1 recognition in chromatin on any chromosome at these early stages, but increasingly labels chromatin after subsequent cell divisions. The early enrichment for H3.3 suggests that, as in other organisms, maternal histone H3.3 is the H3 isoform that contributes to sperm pronuclear chromatin assembly, and oocyte chromatin remodeling, in the zygote [14,15]. Despite this influx of unmodified H3.3 into the chromatin, the original H3K4me2 patterns among the chromosomes are grossly maintained: enrichment of H3K4me2 on the autosomes and oocyte/maternal X (Xm) and striking depletion in Xp chromatin. This pattern persists in early blastomeres through several cell divisions, and in somatic lineages the Xp and Xm eventually become indistinguishable in H3K4me2 enrichment as zygotic transcription increases [68]. In the P-cells, however, the absence of H3K4me2 on the Xp is observed until at least the P3, and may persist into P4 (J. Arico, F. Furuhashi, and WK, unpublished work). As mentioned above, the H3K4me2 on autosomes and the Xm in the P cells is maintained by the MLL proteins [60]. Thus, the genome-wide H3K4me profile in the embryonic germ line, at least at the gross level of analysis afforded by immunofluorescence microscopy, largely matches the profile of the gamete chromatin, and thus also reflects the patterns originating in parental germ cell chromatin. Parental H3K4 methylation patterns established by transcription, like parental H3K36 methylation patterns, persist from the adult germline of one generation into the embryonic germline of the next.

### Transgenerational consequences of germline transcription

Xp linkage is not sufficient to impart heritable repression since active transgenes on the Xp can be expressed in male germ cells, accumulate H3K4me2, and retain the H3K4me2 in the next generation [14]. Conversely, transgenes not linked to the X can exhibit a pattern similar to that of the Xp: transgenes that are repressed in the parental germ cells (of either sex) lack H3K4me incorporation in the adult germ cells, and this pattern persists into the gametes and offspring. Repetitive transgenic versions of ubiquitously expressed genes are strongly subjected to silencing in *C. elegans* germ cells, and those that initially exhibit expression often become stably and heritably repressed in the germ line, even when somatic expression is still evident [69]. This transgenerational repression is very stable once established. It is initiated by RNAi-based mechanisms (discussed below), but appears to be maintained by chromatin-based processes (for example, [69,70]).

Transient expression of such transgenes in germ cells can impart a transgenerationally stable epigenetic memory: H3K4me2 that was incorporated into the transgene chromatin during its expression in germ cells is maintained for many (>20) generations thereafter, irrespective of its lack of germ cell expression in the later generations. Interestingly, this persistent retention of H3K4me in germ cells also correlates with stably increased expression of the transgene in somatic lineages in the later generations [14]. Thus, transcription in germ cells appears to create an epigenetic memory of RNA Pol II activity that is maintained in the germ line for multiple generations, and this memory can affect the level of gene expression in somatic lineages. The instructional role of H3K4me may be analogous to the role of H3K36 methylation’s antagonism to H3K27me, as H3K4me has been noted to have a similarly antagonistic relationship with the repressive modification H3K9 methylation (for example, [71,72]). H3K9 methylation, as discussed below, maintains germline silencing mechanisms initiated by RNA interference (RNAi) pathways, and both play essential roles in heritable modes of gene repression.

Transgenerational regulation of H3K4 methylation in the germline not only correlates with heritable changes in transgene expression as described above, but defects in the machinery that methylates H3K4 can have heritable consequences for endogenous somatic processes. As mentioned, the maintenance of H3K4me2/3 in embryonic germ cells is dependent on conserved Set1/MLL-like complex components, including the SET-2 H3K4 methyltransferase and the conserved core components Wdr5 and Ash2l. Mutations in these components lead to a decrease in H3K4me2/3 maintenance in the early embryo soma and germ line, and loss of Wdr5 and SET-2 cause a defect in germline stem cell maintenance [59-61]. Another phenotype that is observed with depletion of these MLL components in *C. elegans* is prolonged lifespan, and the increase in longevity is dependent on the depletion of these components in the germ line [73]. Amazingly, temporary depletion of SET-2, Wdr5, or Ash2l function within a single generation results in somatic lifespan extension for multiple generations, even after normal function of these factors is restored [74]. The transgenerational aspects of this phenotype, which is also linked to a histone H3K4me2/3 demethylase RBR-2 and its germ cell functions, highlight the importance of correct regulation of this modification as it passages through the germline from each generation to the next.

It is important to note that H3K4 methylation, unlike H3K36me, encounters significant reprogramming in the Z2/Z3 primordial germ cells, thus providing a barrier to transmission of this mark between generations [29]. However, the transgenerational accumulation of H3K4 methylation observed in the *spr-5* mutants, the heritable transgene expression phenotypes, and the transgenerational stability of the aging phenotype in MLL mutants suggests that some level of this modification may not be efficiently (or specifically) reprogrammed in Z2/Z3.

### Adaptive genome immunity and transgenerational repression

In contrast to heritably stable states of gene activation, heritable gene repression is a common and highly studied phenomenon; indeed repression might be considered the default state of a genome encased in chromatin barriers to gene activation. The complex mechanisms that regulate gene expression during development show that overcoming a silent state involves numerous discreet, sometimes energy-dependent steps that culminate in a chromatin state compatible with stable and robust transcription activity. The germline is especially vigilant at preventing spurious transcription, and numerous overlapping activities scrutinize genetic activity to prevent deleterious events, as would be expected for the guardian tissue of the species. As noted above, transgenes frequently become silenced in germ cells, and that silencing becomes essentially permanent in all subsequent generations. This stable, multigenerational repression is clearly under epigenetic control, since such transgenes can still show robust expression in somatic lineages, and/or they can be reactivated if passaged through epigenetic-modifying backgrounds. The heritable repression of transgenic DNA in *C. elegans* has thus been a useful, if sometimes ill-defined, tool for analyzing epigenetic processes in this organism. Introduction of transgenes in worms is most often accomplished by gonadal injection of plasmid DNA, which generates a highly repetitive, nonintegrated (extrachromosomal) array of the injected DNA [75]. These transgenic arrays are subjected to numerous mechanisms that overlap with those that repress transposons and other repetitive genomic elements. Notable among these are those involved with RNA interference (RNAi) pathways.

The regulation of transcriptional repression through aspects of chromatin structure has been known to involve RNAi mechanisms for some time [76]. In many systems, RNAi-related mechanisms can initiate the targeting of repressive chromatin machinery to genomic loci, and subsequent maintenance activities enforce heritable repression [77]. Defects in these mechanisms commonly result in derepression of transposons and repetitive elements, indicating their essential role in genome defense, and nowhere is genome defense arguably more important than in the germline. It is thus not surprising that RNAi-base processes are essential players in the transgenerational inheritance of epigenetic information. In most cases, it appears that RNAi-based mechanisms are focused on the transgenerational inheritance of a repressed state. The highly conserved Piwi-associated small RNA, or piRNA system, which is active in the front lines of genome defense in germ cells, is emerging as a major player in the propagation of repressive epigenetic memory across generations.

piRNAs were first identified as small RNAs that are enriched in the germline of many species, and co-purify with orthologs of the *Drosophila* Piwi protein, a member of the argonaute family of proteins that mediate small RNA-guided processes [78]; reviewed in [79-82]. piRNAs are a hugely abundant class of small RNAs, accounting for tens of thousands of loci, often occurring in clusters on chromosomes in species where they are found [82]. Despite their defined role in transposon defense, transposons are only a subset of known piRNA target loci; that is, the cognate targets of the majority of piRNAs are not repetitive elements. Loss of Piwi and its orthologs in many animals result in partial or complete sterility. The role of piRNA processes in the germline is especially striking in flies and mammals, in which defects in the piRNA pathways yield transposon activation in germ cells and consequential genomic disruption and sterility [reviewed in [80-83]].

In *C. elegans* the piRNA pathway’s function at first glance appears to be less focused on transposons, since depletion of the the *C. elegans* Piwi homolog, *prg-1*, has little effect on transposon mobilization and causes derepression of just one subclass of Tc1/mariner-type DNA elements, Tc3 [84]. However, piRNAs corresponding to other elements have been detected [85]. *prg-1* mutants lack all detectable piRNAs, which are 21 nucleotide RNAs with a characteristic 5′U (21U RNAs). The *C. elegans* genome encodes approximately 30,000 piRNAs, many of which map to unique sequences in two broad clusters on a single chromosome, and are not generally associated with protein coding regions or genes [84-87]. piRNAs that have known targets (for example, a matching sequence on a reporter transgene) generate secondary ‘22G-RNAs’ (22 nucleotide small RNAs with a 5’ guanosine) that match sequences that flank the piRNA target site [88]. These secondary small RNAs are produced by components of the so-called ‘endo-siRNA’ pathways - RNAi paths involving small RNAs produced from endogenous loci (in absence of an external trigger) to target other genes and genomic elements. Interestingly, significant mismatches are tolerated between a piRNA and its targets, which significantly magnifies the potential sequences that can be theoretically targeted by 30,000 piRNAs. However, since the 22G-RNAs are produced from the targets, these secondary effectors can provide a more precise match for the target, refining the specificity of the process. Thus the system, presumably evolved as a genome surveillance process, is an adaptive genome immunity program: it is capable of recognizing an extraordinary range of nucleotide sequences (epitopes), refines that range (clonal selection), and generates an amplified response that narrows the sequences that are efficiently targeted for downstream events. These downstream events can include heritable, multigenerational repression of the targeted gene.

The piRNA pathway intersects with pathways that are required for transgenerationally stable silencing of single-copy transgenes in the germline [88-91] (reviewed in [81]). One of these paths involves a worm-specific argonaut-like protein, named HRDE-1/WAGO-9, which adapts 22G- siRNAs to target H3K9 methylation to genomic regions with antisense complementarity to the 22G-siRNA and repress them [91]. Although small RNAs, such as piRNAs, are required to initiate the silencing, PRG-1 is not required for the heritable maintenance of repression, which has been called RNA-induced epigenetic silencing RNAe; [90]. Other pathways, involving a nuclear RNAi pathway and chromatin interacting and modifying factors are required for the long-term and multigenerational repression [91]. Importantly, these silencing mechanisms involve factors regulating H3K9me3 and its cognate-binding protein, HPL-2, and also MES-4 and the MES/PRC2 components described above [89]. A model has thus emerged in which ‘nonself’ sequences, such as those introduced with transgenic DNA, is recognized by a set of piRNAs with some level of antisense complementarity. This recognition triggers a response that uses the piRNA-identified target to generate a secondary response that is highly specific, amplified, and ultimately recruits chromatin-modifying machinery that solidifies the response via transcriptional repression [92,93]. The chromatin structure whose assembly is directed by these overlapping processes is then maintained in the germline and stable in subsequent generations.

The above model, however, does not explain how nonself is distinguished from self. The extraordinary repertoire of sequences potentially targeted by piRNAs includes mRNAs that are vital for reproduction and embryonic development. The discrimination between these and appropriate targets may be accomplished both by selection against such piRNA sequences, and also by a parallel small RNA pathway that appears to arise from transcripts of genes normally expressed in germ cells. There is evidence for a selection process, since there is a distinct underrepresentation of sequences in germline-expressed loci that are potential target sequences of piRNAs in the genome [85]. In the parallel pathway, a class of 22-G secondary RNAs corresponding to germline-expressed loci and dependent on another worm argonaute homolog, CSR-1, are thought to protect these sequences from piRNA recognition and response [94,95]. Therefore, transgenerational heritability of gene expression in the germ line requires a memory of prior expression in the parental germ cells, otherwise it is targeted as a foreign invader by the genome’s immune surveillance systems, which include piRNAs. A striking example of the requirement for a transcriptional memory was observed by Johnson and Spence: the provision of a maternal transcript corresponding to a sex-determination gene, *fem-1* was required to prevent silencing of wild-type copies of *fem-*1 in the germline of the offspring [96]. The prevention of RNAi-mediated repression through prior transcription conceptually overlaps with the MES-4/PRC2 antagonism that also depends on a memory of transcription and satisfies a general requirement for redundancy in the maintenance of essential biological processes.

## Conclusions

**Figure 6:**
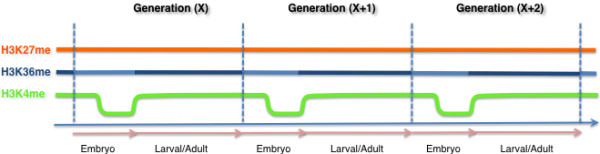
**Transgenerational continuity of histone modifications in the germline.** Continuity in germline chromatin is illustrated for three chromatin modifications: H3K27 methylation (orange), H3K36 methylation (blue), and H3K4methylation (green). Vertical dotted lines demark separate generations; the arrows at the bottom delineate embryonic and postembryonic germ cell stages within each generation. H3K27 methylation levels are maintained by the PRC2 complex at all stages, with an increase in H3K27me3 levels in Z2/Z3 in the embryo (not shown). H3K36 methylation maintenance by MES-4 occurs at all stages, but its maintenance is critical in the embryo (light blue). In postembryonic germ cell stages, co-transcriptional H3K36 methylation by MET-1 occurs (dark blue). H3K4 methylation (green) is maintained in the early embryo by the *C. elegans* MLL complex, but extensive loss/reprogramming of this mark is observed at the birth of the PGCs, Z2/Z3. H3K4 methylation in the postembryonic germ line is then re-established and maintained by a combination of MLL-dependent and MLL-independent/transcription-dependent mechanisms.

Although this story is still incomplete, it is clear that transgenerational inheritance of transcriptional regulation in *C. elegans* involves a complex, overlapping web of epigenetic mechanisms that build a heritable chromatin architecture. That architecture guides gene expression in the germline, which in turn guides chromatin architecture in the zygote and can influence gene expression in somatic lineages. In *C. elegans*, transcription-dependent incorporation of H3K4 and H3K36 methylation, by counteracting H3K9 and H3K27 methylation, respectively, may provide a parental template that is maintained in the offspring and hence across generations via the MLL and MES-4 mechanisms, respectively (Figure 6). This template creates a chromatin signature that may be sufficient to maintain accessibility of promoters to basal transcription machinery, obviating the need for the induction of specific transcription factors to drive germ cell specification at each generation. The maintenance of promoter accessibility may be simply due to prevention of encroachment of repressive histone modifications, that is, by the antagonism of H3K36me versus H3K27m3 and H3K4me versus H3K9me. The overall pattern that results from these and other aspects of chromatin assembly gave rise to functional gametes that generated viable offspring and are therefore proven and worth remembering; that is, it is an epigenetic memory that has been functionally filtered via the requirement for fertility. There is thus a fundamental difference between the regulation of gene expression in germ cells and soma, as perhaps there should be given the germline’s connectivity of generations that span millennia, versus the soma’s single generation relevance. It may be unnecessary for *de novo* specification of germline identity at each turn of the cycle, obviating the need for many of the highly regulated steps that drive developmental transcription programs. Indeed transcription in germ cells substantially differs from that of soma in its requirements for kinases involved in regulating RNA Pol II’s activation, which may indicate some regulatory steps are not required in this lineage [97]. Furthermore, the spatial and temporal control of germline-expressed genes appears to be largely at the post-transcriptional level, with the promoters of these loci limited to simply allowing transcription to occur [98]. Thus, the prevailing models of transcription regulation - based largely on understanding the precise temporal and spatial control required during somatic development - may not apply in a (semi-)continuous lineage such as the germline. All that may be required is the maintenance of a pattern of open promoters in the face of default repression, with the pattern being filtered through, and cemented by, an epigenetic memory of what has successfully produced functional germ cells and viable offspring in previous generations.

The epigenetic patterning of the genomic landscape via co-transcriptional marking in prior generations, along with the RNAs produced, can provide antagonism to the repression mechanisms that operate in the germline and which establish stable states of gene silencing. This antagonism may occur both through H3K4 and H3K36 methylation, which antagonize H3K9 and H3K27 methylation, but also through ‘self’ mRNA-generated small RNAs that antagonize piRNA surveillance. In each system, the robustness of the responses from each side may determine the penetrance of the heritable outcome. Variations in the robustness of either side of the antagonism may contribute to the stochastic nature, and possibly the limits of stability, of many transgenerational phenotypes, such as the limited heritability of increased longevity initiated in Set1/MLL mutants [74]. For example, the loss of H3K4 methylation maintenance in the Set1/MLL mutants may decrease the robustness of this mark in the many metabolic regulatory genes expressed in germ cells. Decreased templating by H3K4me could lead to decreased expression of metabolic loci in the offspring, leading to decreased metabolic activity and heritable longevity, as observed in the study by Greer *et al.*[74]. The reintroduction of wild-type maintenance activity would not immediately return the level of the mark to normal since the Set1/MLL function is predominantly for maintenance of existing levels and may require several generational rounds of reiterative establishment and maintenance to return to normal. The expression may lag until the co-transcriptional H3K4me marking and germline maintenance activities reinforce a return to a steady state that crosses a threshold for normal metabolic activity, and hence normal lifespan. Indeed, changes in metabolic gene expression were predominant among the heritable expression changes observed in the MLL mutant study [74]. Also consistent with this model is the observed role for an H3K4 demethylase, RBR-2, in the heritable longevity [74]. Defective H3K4 *de*methylation at any stage in the germline cycle could substitute for the inefficient maintenance of the mark; that is, H3K4me levels would remain high. Whether the antagonistic RNAi pathways play a role in this heritable process, perhaps through imbalances in production of self-RNAs versus piRNAs, is an open question.

The *C. elegans* epigenetic mechanisms described above and their relevance to transgenerational phenomena in other species is unclear, although the components of each of the pathways covered in this review are highly conserved. All metazoans have MES-4 and PRC2- related enzymes and complexes, and an orthologous MLL complex appears to exist in all eukaryotes, including yeast. The piRNA pathway is likewise a highly conserved, germline specific genome surveillance mechanism, although some aspects of the amplification arm (for example, the role RdRPs) may vary between species. It is also important to note that a major epigenetic pathway, DNA methylation, is not present in worms, and while this simplifies epigenetic analyses in worms, it complicates comparisons with other systems. However, DNA methylation and its maintenance are clearly intertwined with histone modifications, and recent evidence suggests that DNA methylation was only recently lost in the *C. elegans* lineage, as it has been found in a parasitic nematode [99]. Thus, the *C. elegans* modes of intergenerational transfer of epigenetic content, and how that content is screened for retention or removal, may provide yet another useful paradigm for understanding transgenerational processes that contribute to developmental phenotypes in mammals.

## Abbreviations

Ac: acetylation; COMPASS: complex proteins associated with Set1p; GFP: Green fluorescent protein; me2: demethylation; me3: trimethylation; MES: Maternal Effect Sterile; MLL: Mixed lineage leukemia; PRC: Polycomb repressor complex 2; RdRP: RNA-dependent RNA Polymerase; RNAe: RNA-induced epigenetic silencing; RNAi: RNA interference; RNA Pol II: RNA Polymerase II.

## Competing interests

The author declares that he has no competing interests.
